# Prevalence of smoking among national military service recruits in UAE

**DOI:** 10.3389/fpubh.2024.1335545

**Published:** 2024-06-14

**Authors:** Sara Almesmari, Fayeza AlAmeri, Houd Al Shanqaiti, Latifa Al Mansoori, Humariya Heena

**Affiliations:** ^1^Medical Education Department, Zayed Military Hospital, Abu Dhabi, United Arab Emirates; ^2^Department of Family Medicine, Zayed Military Hospital, Abu Dhabi, United Arab Emirates

**Keywords:** smoking, military personnel, tobacco, smoking environment, exercise and smoking, smoking initiation

## Abstract

**Background:**

According to the various screening programs conducted, the prevalence of tobacco use among UAE Nationals is high. A considerable increase is also seen in various forms of smoking is seen among young military men during deployment which results in loss of physical health, less productivity, readiness and increased health care utilization. Also smokers are more likely to develop other addictions and chronic medical conditions.

**Aim:**

To estimate the prevalence of smoking among national military service recruits in the United Arab Emirates and to find its relation with various factors: socio-demographics, lifestyle, comorbidities, and military environment.

**Methods:**

A cross sectional study was conducted amongst national service recruits selected by random stratification through a self-administered anonymized questionnaire which was distributed to a final sample of 369 patients. Data was analyzed using SPSS version 16. Chi square, percentage and frequencies were used to present the data where applicable. A *p* < 0.05 was considered to be significant.

**Results:**

The prevalence of smoking among national military service recruits was 41.6%. As the level of education increased the prevalence of smoking decreased. Smokers with insufficient income, divorced or widowed recruits tend to smoke more as against single and married recruits. Smoking rates were decreased in those who exercise regularly. There was a significant relationship between smoking status and chronic diseases. Smoker gatherings inside military campus encouraged initiation of smoking or its continuity. About half of the smokers were not satisfied with environment at military facilities.

**Conclusion:**

Smoking has high prevalence among national service military recruits. For this reason, a goal directed future plan toward screening of smokers among recruits and assigning them to smoking cessation clinics and educational seminars prior to joining the national service is the need of the hour.

## Introduction

Smoking is the most preventable cause of death and it is predicted that in the next 20 to 30 years, 10 million people annually will die from tobacco smoking with 70%−80% of these deaths occurring in the developing countries (1).

The overall pooled prevalence of current cigarette and water pipe smoking among adults in 17 Middle Eastern countries is 17.41% and 6.92%, respectively (2). According to a 2017 WHO report, 14 million adult smokeless tobacco users live in the Eastern Mediterranean region, of whom 11 million are male and 3 million are female (3).

Tobacco use and exposure in the Emirate of Abu Dhabi is relatively high, with 36% of men reporting tobacco use and nearly 30% of women reporting exposure to tobacco at home. Poly-use is common among men, with more than a third of men reporting dual or poly-use of tobacco (4).

According to 2011 data from the screening program, tobacco use was estimated to be 21.6% among UAE Nationals with Midwakh or Dokha followed by cigarette as the most common form and water-pipe smoking being less common (5).

Smokeless tobacco consumption increases considerably in young military men who are deployed to far off locations (6). According to the U.S. Centers for Disease Control and Prevention (CDC), approximately one in four of all active-duty military service members and more than half of male veterans aged 18-25.5 years' currently smoke. Also, tobacco use costs the military in lost productivity and healthcare expenses. Many young military personnel use both cigarettes and smokeless tobacco and commonly initiate tobacco use after they complete basic training during which tobacco use is prohibited. The prevalence rates of smoking among military personnel show wide variations among countries. Very high rates have been reported in the certain European armies with more than half being current smokers (7).

Recent studies have shown that the prevalence of cigarette smoking increases during military service and is higher in young military conscripts than in the general population. A 2016 US Department of Defense study found that 38 percent of current smokers in the military began smoking after joining. Among junior enlisted personnel, about 30 percent report current cigarette smoking after joining the military (9, 10).

A meta-analysis showed that, almost 20% of adults in the Middle East were cigarette smokers and 7% were water pipe users with Iraq, Cyprus, and Palestine on top while the lowest prevalence was in Oman and Bahrain (11, 12). Socioeconomic status and different customs and cultures may explain these differences in prevalence.

The popularity of cigarette smoking is still a public health problem among adults, particularly in men in Middle East countries. About 30% of esophageal and lung cancers in this region are attributed to cigarette smoking (13). Keeping in view the importance of tobacco consumption due to its role as a modifiable risk factor in various diseases, and the paucity of studies in this field in the military, we undertook this study to understand the burden of smoking and enabling of specific military environment toward its' initiation.

## Materials and methods

### Study design and participants

A total of 379 male recruits reporting to a regimental Center hailing from different camps around the emirate of Abu Dhabi in the age group 17–23 years were approached for the study.

### Measurements

A structured anonymous questionnaire on smoking/tobacco use was administered. The content and format of this questionnaire was derived from WHO published guidelines and previous research (1–3) and pretested in a pilot study. The subjects were asked to complete the questionnaire which was self-administered, so as to reduce under-reporting of tobacco use. Questions were related to types of smoking/tobacco use, frequency, parental tobacco use, peer pressure and knowledge of harmful effects of tobacco use.

An established criteria were used to define smoking status (3). A nonsmoker was defined as one who has never smoked. Experimental smoker was the one who tried smoking only once. For occasional smoker, smoking frequency was less than once a week. A light smoker was the one who smoked 1–6 cigarettes/bidis in a week. A regular smoker smoked more than 6 cigarettes/bidis in a week. A heavy smoker smoked 10–19 cigarettes per day. A very heavy smoker smokes >19 cigarettes per day. Habit of smokeless tobacco was elicited by asking about habituation to different types of smokeless tobacco such as tobacco in pan, chewing, pan masala, etc.

Parental tobacco use was defined as habit of smoking/smokeless tobacco use by either or both parents. Peer pressure was decided by response to questions such as: (a) whether the recruit used tobacco on persuasion of close friends (b) as imitation of the habit of close friends or (c) to impress close friends. Affirmative answer to any of these was taken as peer pressure.

### Study design

This cross-sectional survey was conducted among national service recruits in UAE.

Sample size and population: Out of 4,509 males who joined the mandatory national service from November 2021 to April 2022 in the UAE, a representative sample of 379 recruits were stratified by national services camps to fill out the questionnaire. The national services camps were stratified based on fitness and code named into fit (AA and LW) and unfit (SL and AM). However, females were excluded from the study as the national military service is not mandatory for them.

### Investigation tool

A structured and self-filling questionnaire was developed with four sections. Section one described the social demographic characteristics of the study population while the section second contained questions about health and lifestyle. The third and fourth sections had questions about the military national service environment and smoking, respectively. People who either neglected or refused to fill in the necessary data were excluded from the study.

### Data collection

Data was collected from January to March. Questionnaires were distributed by two physician colleagues who visited the selected two National service campuses (known as SL and AA). The confidentiality of the participants was maintained as the anonymous questionnaire required no identifying data. They were collected in boxes once the participants had completed the questionnaire. A written informed consent was signed by all participants.

### Data analysis

Data analysis was done using SPSS version 16. All categorical variables were presented as frequency and percentage. Continuous variables were expressed as Mean ± SD. Chi-square test was used to determine significant associations between categorical variables. A two-sided *p* < 0.05 was considered statistically significant.

### Ethical consideration

The study was approved by the Ethics and Research committee at Zayed Military Hospital, Abu Dhabi.

## Results

Out of the total 4,509 male recruits, a final sample of 369 patients was obtained after excluding some missing data. The response rate for the study for each characteristic was different probably due to the hesitancy of the recruits to answer sensitive questions according to them. The sociodemographic characteristics are depicted in Table 1. Among the population studied the majority were aged between 20 and 30 years (77%). Most of them were single (64.8%) with no children (75.8%). 59.5% of our participants were highly educated. Majority were employed with the government (74.9%) and about half (49.9%) had sufficient income. Majority (76.9%) of our participants were healthy with only 6.3% having respiratory diseases. 80.2% of the study sample had no psychiatric illness with anxiety (13.5%) being more common than depression or OCD. Most of our population had normal body weight (67.9%) while a small number (18.8%) were overweight or obese. Most of our recruits ate a balanced diet (70.6%). 89.7% consumed fast food and soft beverages. The most common exercise practiced by our recruits was walking (50.8%) and the least common was cycling (9.3%). A total 55.2% of our participants exercised 3-5 times weekly. The prevalence of alcohol consumption and drug abuse among our participants was 2.8% and 1.7% respectively. 66.6% of our population was satisfied with the military facilities and 86.1% said that sleeping hours were not enough. Regarding military exercises, 71.2% of our participants showed satisfaction with the military trainer attitude and 44.3% said military exercises were tough. In respect to smoker gatherings inside military campuses, 37% considered it as a factor of smoking initiation and continuation.

**Table 1:** Socio-demographic characteristics of the study sample.

**Characteristics**	**Frequency**
**Age (*****n** =* **369)**	
<20 years	22 (6)
20–30 years	284 (77)
>30 years	63 (8)
**Marital status (*****n** =* **369)**	
Single	239 (64.8)
Married	127 (34.4)
Divorced/Widowed	3 (0.8)
**Children (*****n** =* **368)**	
Yes	89 (24.2)
No	279 (75.8)
**Education (*****n** =* **365)**	
Secondary school	148 (40.5)
University and above	217 (59.5)
**Employment (*****n** =* **366)**	
Government	274 (74.9)
Self-employed	14 (5.2)
Unemployed	63 (17.2)
Student	10 (2.7)
**Income (*****n** =* **361)**	
Insufficient	81 (22.4)
Sufficient	180 (49.9)
Sufficient and saving	100 (27.7)
**Chronic disease (*****n** =* **364)**	
None	280 (76.9)
HTN	9 (2.5)
DM	6 (1.6)
CVD	5 (1.4)
Respiratory diseases	23 (6.3)
Others	58 (15.9)
**Clinically diagnosed Psychiatric**	
Disorders (*n =* 364)	
None	292 (80.2)
Depression	24 (6.6)
Anxiety	49 (13.5)
Obsessive compulsive disorder	34 (9.3)
**Body weight (*****n** =* **368)**	
Underweight	49 (13.3)
Normal	250 (67.9)
Overweight	65 (17.7)
Obese	4 (1.1)
Balanced diet	255 (70.6)
Fast food	138 (38.2)
Soft drinks	186 (51.5)
**Type of exercise**	
Walking (*n =* 248)	126 (50.8)
Running (*n =* 248)	93 (37.5)
Cycling (*n =* 247)	23 (9.3)
Swimming (*n =* 246)	31 (12.6)
Gym (*n =* 247)	97 (39.3)
Other exercise (*n =* 247)	49 (19.8)
**Exercise frequency (*****n** =* **306)**	
Daily	39 (12.7)
3-5 times weekly	169 (55.2)
Once weekly	98 (32)
Alcohol (*n =* 362)	10 (2.8)
Drugs (*n =* 362)	6 (1.7)
Satisfaction with the facilities (*n =* 376)	241 (66.6)
Sleep adequacy (*n =* 366)	51 (13.9)
Satisfaction with the Trainer's attitude (*n =* 364)	259 (71.2)
Toughness of exercises (*n =* 366)	162 (44.3)
Smoker gatherings initiating smoking (*n =* 362)	134 (37)

The prevalence of smoking among national service recruits was 41.8% (Table 2). Between the types of smoking, pipe smoking (84.5%) was the highest while E-cigarette smoking (5.8%) was the lowest. With respect to smoking frequency, the majority smoked <10 cigarettes daily (67.2%), pipe >3 refills daily (68.7%) and water pipe /e-cigarettes <5 times daily (69.7%). Among smokers, the majority had signs of eye opener within 5 min (44.8%). During national service training, 8.7% of the recruits started smoking while 91.3% were already smokers. Regarding smoker gatherings inside military campus, 27.8% initiated or were encouraged for continuity of smoking while 68.4% of non smokers agreed to this statement. A total 53.1% of smokers experienced withdrawal symptoms during military course. In regards to quitting, 53.8% of smokers thought of quitting seriously, 71.3% had tried quitting before, 12.6% tried quitting drugs during the training, 40.5% had quitting relapses. Stress (46.6%) was considered the main reason that prevented them from quitting. A total 36.3% of recruits noticed their colleagues started smoking during the national service course.

**Table 2:** Smoking characteristics of the study sample.

**Characteristics**	**Frequency (%)**
Smoking status (*n =* 366)	153 (41.8)
Cigarettes (*n =* 155)	62 (40)
Pipe (*n =* 155)	131 (84.5)
Water pipe (*n =* 155)	39 (25.2)
e-cigarettes (*n =* 155)	9 (5.8)
Cigarettes frequency (*n =* 58)	
<10 daily	39 (67.2)
10-20 daily	12 (20.7)
>20 daily	7 (12.1)
**Pipe frequency (*****n** =* **134)**	
Once daily	6 (4.5)
2-3 daily	36 (26.9)
>3 daily	92 (68.7)
**Water pipe frequency (*****n** =* **33)**	
<5 daily	23 (69.7)
5-10 daily	5 (15.2)
>10	5 (15.2)
**Eye opener (*****n** =* **125)**	
Within 5 min	56 (44.8)
6-30 min	38 (30.4)
31-60 min	16 (12.8)
After 60 min	15 (12)
**Smoking initiation (*****n** =* **150)**	
Before national service	137 (91.3)
During national service	13 (8.7)
Smoking gatherings in military campus (*n =* 162)	45 (27.8)
Withdrawal Symptoms (*n =* 147)	78 (53.1)
Smoking QUITTING intention (*n =* 156)	84 (53.8)
Smoking quitting trial (*n =* 157)	112 (71.3)
Quitting drugs (*n =* 159)	20 (12.6)
Quitting relapse (*n =* 148)	60 (40.5)
Social gatherings barrier (*n =* 149)	50 (33.6)
Withdrawal barrier (*n =* 148)	31 (20.9)
Stress barrier (*n =* 148)	69 (46.6)
Craving barrier (*n =* 148)	51 (34.5)
Other barriers (*n =* 148)	35 (23.6)
NS colleagues start smoking (*n =* 353)	128 (36.3)

Participants with secondary school level of education tend to smoke more (48.3%) in comparison to university level educated participants (36.6%) (Table 3). Secondary school age recruits smoked more cigarettes (51.4%) as compared to others. Smokers with insufficient income tend to smoke pipe (35.9%) more than those with sufficient income (15.8%) and saving income (32.4%). Divorced/widowed recruits smoke more water pipe in contrast to singles (24.7%) and married (22.8%). Half of the divorced participants smoked e-cigarettes. These results are statistically significant (*P* < 0.05). However, there is no statistically significant relationship between smoking status and age, type of employment, and recruits without children (*P*-value of 0.2, 0.4, and 0.5 respectively).

**Table 3:** Relation between smoking and sociodemographic characteristics.

	**Smoking status**				**Cigarettes**			**Pipe**		
	**Yes**	**No**	**Ex-smoker**	***P*** **-value**	**Yes**	**No**	***P*** **-value**	**Yes**	**No**	***P*** **-value**
**Age**										
<20	9 (42.9)	12 (57.1)	0 (0)	0.2	5 (55.6)	4 (44.4)		9 (100)	0 (0)	
20-30	109 (39.2)	155 (55.8)	14 (5)		42 (37.8)	69 (62.2)		91 (82)	20 (18)	
>30	31 (51.7)	28 (46.7)	1 (1.7)		12 (38.7)	19 (61.3)	0.57	27 (87.1)	4 (12.9)	0.3
**Education**				<0.05			<0.05		65 (82.3)	0.5
Secondary school	69 (48.3)	71 (49.7)	3 (2.1)		36 (51.4)	34 (48.6)		60 (85.7)		
University	78 (36.6)	123 (57.7)	12 (5.6)		22 (27.8)	57 (72.2)		65 (82.3)	14 (17.7)	
**Employee**										
Government	104 (39)	152 (56.9)	11 (4.1)		36 (34)	70 (66)		90 (84.9)	16 (15.1)	
Self-employed	9 (47.4)	10 (52.6)	0 (0)	0.4	6 (66.7)	3 (33.3)	0.1	7 (77.8)	2 (22.2)	0.9
Unemployed	30 (50)	26 (43.3)	4 (6.7)		14 (46.7)	16 (53.3)		26 (86.7)	4 (13.3)	
Student	5 (50)	5 (50)	0 (0)		3 (60)	2 (40)		4 (80)	1 (20)	
**Income**										
Insufficient	39 (49.4)	35 (44.3)	5 (6.3)		19 (48.7)	20 (51.3)		34 (87.2)	5 (12.8)	
Sufficient	74 (42.8)	96 (55.5)	3 (1.7)	0.07	25 (32.9)	51 (67.1)	0.2	64 (84.2)	12 (15.8)	0.6
Saving	34 (34.3)	60 (60.6)	5 (5.1)		14 (41.2)	20 (58.8)		27 (79.4)	7 (20.6)	
**Marital**										
Single	92 (39.5)	131 (56.2)	10 (4.3)		40 (43)	53 (57)		81 (87.1)	12 (12.9)	
Married	56 (45.5)	63 (51.2)	4 (3.3)	0.7	18 (31.6)	39 (68.4)	0.08	45 (78.9)	12 (21.1)	0.34
Divorced	2 (66.7)	1 (33.3)	0 (0)		2 (100)	0 (0)		2 (100)	0 (0)	
**Children**										
Yes	40 (46.5)	43 (50)	3 (3.5)	0.5	15 (37.5)	25 (62.5)	0.7	33 (82.5)	7 (17.5)	0.7
No	109 (40.1)	152 (55.9)	11 (4)		45 (40.5)	66 (59.5)		94 (84.7)	17 (15.3)	

There is significant relationship between smoking exercise, and alcohol (*P*-value 0.003, 0.007 respectively) (Table 4). On the other hand, weight, balanced diet, fast food and drug use have no significant relation with smoking status. There is a statistically significant relationship between smoking status and chronic diseases; 52.4% in comparison to non-smokers (*P*-value = 0.04). Regarding Psychiatric diseases, depression, anxiety and obsessive-compulsive disorder results showed no significant relationship with smoking status. Table 4 emphasizes on the relationship between smoking and satisfaction with the military environment, which includes satisfaction with military facilities, as 51.2% of smokers were not satisfied as compared to 44.4% of non-smokers who were not satisfied. This result is statistically significant with *p* = 0.035. A total 37.6% of smokers thought that military training was tough in comparison to 58.5% of non-smokers who thought that military training was tough (*p* = 0.025). A total 24.8% of smoker claimed that the gatherings of smokers encouraged smoking in comparison with 68.4% non-smokers (*p* < 0.05). There was no statistically significant relationship between smoking and sleep duration and rigid military exercise regimen (*p* = 0.886 and *p* = 0.450 respectively). Notably, 37.5% who were self-employed started smoking after joining national service in the military and 92.2% who were employed by the government were smokers before joining the national service (*p* = 0.033).

**Table 4:** Relationship between smoking and lifestyle, comorbidities, military service.

	**Smoking**			
	**Yes**	**No**	**Ex-smoker**	***P*** **-value**
**Weight**				
Underweight	22 (47.8%)	24 (52.2%)	0 (0%)	0.371
Normal	103 (42.2%)	130 (53.3%)	11 (4.5%)	
Overweight	21 (32.8%)	40 (62.5%)	3 (4.7%)	
Obese	3 (75%)	1 (25%)	0 (0%)	
**No meat**				
Yes	8 (53.3%)	7 (46.7%)	0 (0%)	0.496
No	136 (40.5%)	186 (55.4%)	14 (4.2%)	
**No fish**				
Yes	27 (56.2%)	18 (37.5%)	3 (6.2%)	0.031
No	117 (38.6%)	175 (57.8%)	11 (3.6%)	
**No chicken**				
Yes	3 (50%)	3 (50%)	0 (0%)	0.822
No	141 (40.9%)	190 (55.1%)	14 (4.1%)	
**Eat everything**				
Yes	94 (38.1%)	145 (58.7%)	8 (3.2%)	0.08
No	50 (48.1%)	48 (46.2%)	6 (5.8%)	
**Fast food**				
**Yes**	49 (30%)	81 (59.6%)	6 (4.4%)	0.317
**No**	95 (44.2%)	112 (52.1%)	8 (3.7%)	
**Exercise**				
Yes	75 (35.9%)	120 (57.4%)	14 (6.7%)	0.003
No	78 (50.3%)	75 (48.8%)	2 (1.3%)	
**Alcohol**				
Yes	9 (90%)	1 (10%)	0 (0%)	0.007
No	142 (40.5%)	193 (55%)	16 (4.6%)	
**Drugs abuse**				
Yes	4 (66.7%)	1 (16.7%)	1 (16.7%)	0.102
No	146 (41.1%)	194 (54.6%)	15 (4.2%)	
**Chronic disease**				
Yes	43 (52.4)	35 (42.7)	4 (4.9)	
No	103 (37.9)	159 (58.5)	10 (3.7)	
**DM**				
Yes	0 (0%)	5 (100%)	0 (0%)	0.1
No	146 (41.8%)	189 (54.2%)	14 (4%)	
**HTN**				
Yes	5 (55.6)	4 (44.4)	0 (0)	0.6
No	141 (40.9)	190 (55.1)	14 (4.1)	
**Respiratory diseases**				
Yes	14 (60.9)	9 (39.1)	0 (0)	0.1
No	132 (39.9)	185 (55.9)	14 (4.2)	
**CVD**				
Yes	4 (80)	1 (20)	0 (0)	0.2
No	141 (40.5)	193 (55.5)	14 (4)	
**Psychiatric diseases**				
Yes	35 (50)	34 (48)	1 (1.4)	0.1
No	112 (39.4)	159 (56)	13 (4.6)	
**Depression**				
Yes	13 (54.2)	10 (41.7)	1 (4.2)	0.4
No	134 (40.6)	183 (55.5)	13 (3.9)	
**Anxiety**				
Yes	21 (24.7)	25 (53.2)	1 (2.1)	0.7
No	126 (41)	168 (54.7)	13 (4.2)	
**OCD**				
Yes	14 (43.8)	17 (53.1)	1 (3.1)	0.9
No	133 (41.3)	176 (54.7)	13 (4)	
**Satisfaction with military facilities**				
Yes	89 (37.1%)	140 (58.3%)	11 (4.6%)	0.035
No	62 (51.2%)	54 (44.6%)	5 (4.1%)	
**Adequate sleep**				
Yes	20 (39.2%)	29 (56.9%)	2 (3.9%)	0.886
No	133 (42.4%)	167 (53.2%)	14 (4.2%)	
**Rigid military training regimen**				
Yes	97 (37.6%)	151 (58.5%)	10 (3.9%)	0.025
No	54 (51.4%)	45 (42.9%)	6 (5.7%)	
**Tough military exercise regimen**				
Yes	72 (44.4%)	80 (49.4%)	10 (6.2%)	0.45
No	80 (39.8%)	115 (57.2%)	6 (3%)	
**Smokers gatherings encouraged smoking**				
Yes	33 (24.8%)	91 (68.4%)	9 (6.8%)	
No	120 (52.6%)	101 (44.3%)	7 (3.1%)	

96.6% of married recruits were already smokers before joining military training however all divorced recruits asserted that they started smoking after joining military (*p* < 0.05). Smoking initiation was not related to age, education, income, and children (*p* = 0.239, *p* = 0.446, *p* = 0.122, *p* = 0.063, and *p* = 0.477) respectively.

A total 97.9% of smokers before joining military national service were also fast food consumers in comparison to 87.4% non smokers before the service (*p* = 0.04) (Table 5).

**Table 5:** Relationship between smoking initiation before and after joining the military service with demographics and lifestyle.

**Smoking initiation**	**Before NS**	**During NS**	***P* value**
**Weight**			
Underweight	21 (95.5%)	1 (4.5%)	0.4
Normal	91 (91%)	9 (9%)	
Overweight	19 (90.5%)	2 (9.5%)	
Obese	2 (66.7%)	1 (33.3%)	
**Healthy Diet**			
Yes	83 (89.2%)	10 (10.8%)	0.3
No	46 (93.9%)	3 (6.1%)	
**Fast Food**			
Yes	46 (97.9%)	1 (2.1%)	0
No	83 (87.4%)	12 (12.6%)	
**Exercise**			
Yes	62 (89.9%)	7 (10.1%)	0.2
No	70 (94.6%)	4 (5.4%)	
**Alcohol**			
Yes	7 (87.5%)	1 (12.5%)	0.6
No	123 (92.5%)	10 (7.5%)	
**Drugs**			
Yes	2 (66.7%)	1 (33.3%)	0.1
No	128 (92.8%)	10 (7.2%)	

Regarding recreational drug users, 66.7% were smokers before joining military national service and 33.3% started smoking during national service (*p* = 0.09) (Table 5).

## Discussion

In our study, the prevalence of smoking was high. Usage of pipe smoking was the highest while usage of E-cigarettes was the lowest. A lower prevalence rate of 39% has also been reported among USA soldiers while UK has one of the lowest rates, with 31.3% of soldiers being current smokers (14, 15). The prevalence rate in our study also appears to be higher as compared to a study conducted on Saudi Arabian Army from all military regions (16). The higher prevalence of smoking among the UAE national service recruits is probably due to the high stress level from multiple obligatory tasks along with an unstable social life. Notably, in Saudi Arabia recruits join the army voluntarily and chose it as their job whereas our study included recruits who had joined the national military service as an obligatory requirement.

Our study shows that a fraction of smokers started smoking during national service training and a considerable number of them noticed that their colleagues had started smoking during the training (17). This is similar to what was reported in a study in KSA where many young military personnel take to tobacco use after they complete their basic military training (8). Initiating smoking during military courses could be because of the stress that recruits experience plus the lack of support and health education about smoking. The prevalence of smoking is less in married recruits with higher education level and sufficient income which is similar to other studies. This is because those who are married, educated and have sufficient income are more mature, with stability in their relationships and presumably more aware about the dangers of smoking.

Working with low income is a positive predictor of current smoking while older age, higher education, higher rank and marital status were protective factors for smoking. In one study, 24 percent of military personnel smoked, compared to 19 percent of the civilian population with 25% men and 17.8% women among them (18). There is an inverse relationship between smoking in the military and pay grade; the lowest paid military personnel are more likely to be smokers than the highest paid officer.

Military personnel who smoke are less productive and do not perform as well on physical fitness tests when compared to non-smoking personnel. In a study on how smoking status and being overweight predict fitness levels among a military population, smoking was a stronger and more consistent predictor of fitness for duty (including physical and mental health) than being overweight (19).

The effect of smoking on military personnel is damaging. Apart from the associated organic physical disorders with more hospitalizations, smoking has a negative impact on fitness and productivity. Given these harmful effects and the consequent considerable costs, the increasing trend in smoking during the last decade is of concern.

Smokers have been shown to have lower mental capacities and fitness for duty, less readiness, substance abuse, and legal problems. In regards to the relation between alcohol consumption and smoking, our study showed that smokers are more likely to consume alcohol (20). This relationship was also emphasized by a study that showed that alcohol dependence is associated with increased vulnerability to smoking initiation, onset and persistence of nicotine dependence. This is because smokers tend to get more addicted to other substances like alcohol as compared to non-smokers (21). Moreover, our study showed that those who exercise regularly are less likely to be smokers which is reiterated by existing literature (22). This is due to the increased awareness of the role of regular exercise on overall health and the importance of maintaining it, by avoiding harmful practices especially smoking.

Also, smoker recruits tend to have chronic diseases more than the non-smokers. And this is due to the fact that smoking is associated with and causes many chronic diseases like hypertension (23).

Regarding military environment, our study showed that those who smoke are not satisfied with the military facilities more than the non-smokers. This could be due to the fact that smoking is prohibited inside the facilities and hence the dissatisfaction of only the smokers.

The study also showed recruits who started smoking during national service are mainly divorced or widowed and self-employed. This is because the psychological stress and instability that these groups of recruits' experience is more than others (24).

We also found that all the negative life style traits like eating fast food, not exercising regularly, being obese or drinking alcohol or the rigid military environment (tough exercises and inadequate sleep) did not contribute to initiation of smoking during national service. Most of recruits started smoking before joining the national service regardless of life style, habit or military environment regimen.

## Conclusion

The prevalence of smoking among national service recruits is significant to warrant attention as most of them are young single healthy adults with no chronic diseases and had started smoking before joining military course training. Our study implied that performing regular exercises, avoiding alcohol, providing a healthy military environment and discouraging gatherings encouraging smoking can act as deterrents for smoking initiation during national military service training.

## Limitation

The limitations in this study were limited regional military studies of prevalence in the UAE to compare our results with. Some participants did not answer the questionnaire completely due to unknown reasons.

## Recommendation

Future plan goals directed toward proper screening for each recruit, assigning them to smoking cessation clinics and conducting educational seminars prior to joining national service should be initiated.

## Data availability statement

The datasets presented in this article are not readily available because the datasets belong to the third party (Zayed Military Hospital) and need special permission for access.

## Ethics statement

The study was approved by the Ethics and Research Committee, Zayed Military Hospital, Abu Dhabi, UAE. The study was conducted in accordance with the local legislation and institutional requirements. The participants provided their written informed consent to participate in this study.

## Author contributions

SA: Investigation, Project administration, Supervision, Writing – review & editing. FA: Supervision, Investigation, Project administration, Resources, Visualization, Writing – review & editing. HA: Conceptualization, Data curation, Methodology, Software, Writing – original draft. LA: Formal analysis, Investigation, Methodology, Software, Writing – original draft. HH: Resources, Supervision, Validation, Visualization, Writing – review & editing.
